# Perfectionism and Depression: Vulnerabilities Nurses Need to Understand

**DOI:** 10.1155/2011/858497

**Published:** 2011-05-29

**Authors:** Sherri Melrose

**Affiliations:** Centre for Nursing and Health Studies, Faculty of Health Disciplines, Athabasca University, Athabasca, AB, Canada T9S 3A3

## Abstract

Striving for excellence is an admirable goal. Adaptive or healthy perfectionism can drive ambition and lead to extraordinary accomplishments. High-achieving people often show signs of perfectionism. However, maladaptive, unhealthy, or neurotic perfectionism, where anything less than perfect is unacceptable, can leave individuals vulnerable to depression. In both personal and professional relationships, nurses need to understand how accepting only perfection in self and others is likely to lead to emotional distress. This paper reviews perfectionism as a personality style, comments on perfectionism and high achievement, discusses vulnerabilities to depression, identifies how to recognize perfectionists, and presents balancing strategies perfectionists can implement to lessen their vulnerability to depression.

## 1. Introduction

*Striving for excellence motivates you; striving for perfection is demoralizing.*

*(Harriet Braiker)* *(Harriet Braiker)*

Striving for excellence in any endeavor is generally considered an admirable way of thinking. Setting high expectations and conscientiously striving to achieve difficult but attainable goals usually leads to feelings of satisfaction. However, when thinking shifts towards perfectionism, defined as “setting excessively high standards of performance in conjunction with a tendency to make overly critical self-evaluations” [1, page 450], emotional distress, particularly negative affect and depression, often results [2–9]. Perfectionism differs from a healthy attitude of striving to achieve. Maladaptive or neurotic perfectionism impacts individuals from all walks of life, and yet the construct is seldom addressed in the nursing literature. This paper reviews perfectionism as a personality style, comments on perfectionism and high achievement, discusses vulnerabilities to depression, identifies how to recognize perfectionists, and presents balancing strategies perfectionists can implement to lessen their vulnerability to depression. 

According to the Diagnostic and Statistical Manual of Mental Disorders DSM-IV-TR, major depression is defined by depressed mood, markedly diminished interest or pleasure, significant weight loss or gain, insomnia or hypersomnia, psychomotor agitation, fatigue, feelings of worthlessness or guilt, diminished ability to think or concentrate, and recurrent thoughts of death [10]. Symptoms of depression vary, and while some can present as mild responses to distressing life events, others are severely disabling and persistently distort how individuals' view themselves and the world around them. Understanding these distorted views is not straightforward. However, research revealing positive correlations between depressive symptoms and perfectionism begins to offer important insights. 

## 2. Perfectionism as a Personality Style

Perfectionism has been conceptualized both as a stable personality trait, where individuals habitually engage in the same patterns of behavior [11] and a thinking style, or the ways in which individuals think about those behaviours [12]. A perfectionist personality style is not viewed as a disorder, but rather as a vulnerability factor in producing depression and other psychological problems in adults, adolescents, and children [13]. The following explanation of three subtypes or components of a perfectionist personality style begins to illustrate how this worldview has personal as well as social ramifications for afflicted individuals. 

Hewitt and Flett [11] viewed perfectionism as a multidimensional construct with three elements: self-oriented perfectionism, other-oriented perfectionism, and socially-prescribed perfectionism. First, self-oriented perfectionism is an intrapersonal dimension that involves requiring perfection of oneself, constantly striving to achieve unrealistically high standards, and critically evaluating one's own performance. Second, other-oriented perfectionism is an interpersonal dimension that involves unrealistic expectations and harsh evaluation of others. Third, socially-prescribed perfectionism is also an interpersonal dimension. However, socially prescribed perfectionism involves the perception that perfectionist standards are held by important persons in one's life, and that these important others expect perfection and will evaluate performance critically [6, 14]. Thus, those with a perfectionist personality style can demand perfection in themselves, in others, or they can believe others will accept only perfection. 

The pathways where any one of these dimensions of perfectionism can heighten an individuals' vulnerability to depression becomes clear when we consider the magnitude of dissatisfaction they live with everyday. “Striving to be flawless” [14] leaves individuals persistently feeling as though they have failed, that nothing they do will ever be good enough and that mistakes are unacceptable. 

## 3. Perfectionism and High Achievement

On the other hand, although individuals with perfectionist personality styles are vulnerable to experiencing feelings of hopelessness and depression, they can also be well served by these tendencies. Hamachek [12] posited a seminal distinction between two forms of perfectionism. He described an adaptive, healthy positive form labeled “normal,” where individuals engage in “relaxed and careful” (page 28) pursuit of activities and evaluate themselves against high but reasonable self-standards. In contrast, he also described a maladaptive, unhealthy, negative form labeled “neurotic,” where individuals engage in “tense and deliberate” (page 28) pursuit of unreasonable expectations. While adaptive perfectionists derived pleasure from their striving, maladaptive perfectionists “never seem to do things good enough to warrant that feeling” [12, page 27].

Focusing on adaptive perfectionism, Stoeber and Otto's [15] paper demonstrated that perfectionist strivings were associated with positive attitudes and that healthy perfectionists demonstrated higher levels of positive characteristics than either unhealthy perfectionists or nonperfectionists. Stoeber and Otto's findings suggested that self-oriented perfectionist strivings were positive, as long as tendencies to become overly concerned about mistakes and negative evaluations by others were not present. 

Further, adaptive perfectionism has been linked to conscientiousness [16], overcoming procrastination [17] and self-efficacy [18]. Perfectionist strivings can be associated with higher satisfaction with life [19]. Those with adaptive perfectionism tend to have high self-esteem and are relatively immune to the long-term detrimental effects of perceived failures [20].

Gifted people [21] and high achieving athletes [22] often show signs of perfectionism. Adaptive perfectionists achieve academically [20, 23] and frequently have high Grade Point Averages [24, 25]. 

Adaptive perfectionism may even help people live longer. Positive associations between perfectionist young adults and better physical health [26], as well as less engagement in health-risk behaviours such as smoking and drinking [27], have been identified. Fry and Debat's [28] investigation of seniors newly diagnosed with diabetes revealed that perfectionist seniors lived longer than their less exacting peers who faced the same challenges. Fry and Debat's work invites other nurse researchers to consider how a perfectionist outlook might foster clients' ways of thinking about managing their illnesses.

## 4. Vulnerability to Depression

A key link between maladaptive perfectionism and depression is how critically one evaluates oneself when expected goals are not achieved. When those with maladaptive perfectionist personality styles show patterns of concern over mistakes and consistent doubts about their actions, they can be identified as “clinically significant perfectionists” [29]. Although perfectionist personality styles can foster high achievement, clinically significant perfectionism renders individuals vulnerable to depression and becoming inflexible towards changing their way of thinking, despite the negative impact that the pursuit of perfectionism has on their quality of life [9]. 

A critical health concern with clinically significant perfectionists is the elevated risk of suicide. Perfectionism can play a role in suicide [3], and socially prescribed perfectionism correlates positively with perfectionist thinking [30, 31]. Women with eating disorders [32], depression during pregnancy [33], postpartum depression [34], and both eating disorders and postpartum depression [35] are all particularly vulnerable to depression when their perfectionism becomes clinically significant. Similarly, given that perfectionism is closely associated with obsessive compulsive symptoms [36], when perfectionist tendencies exacerbate, the strength of that association is substantially greater and depression worsens [37]. 

Perfectionism is believed to be linked to depression because perfectionists base their self-worth on being successful and on the need to be actively working toward their goals. Therefore, with self-worth contingent only on fully achieving goals, depressive symptomatology is bound to occur when some goals are not met [38]. Likewise, self-esteem lowers when perfectionists' goals are not met [3, 39–42]. Perfectionism has been found to correlate highly with internalized shame [2]. 

Perfectionists engage in high levels of brooding and ruminating [43], where they go over and over their mistakes. They live with a constant expectation of negative consequences [44]. In summary, clinically significant perfectionists have little respite from sustained feelings of decreased self-worth, low self-esteem, shame, rumination about mistakes, and expecting only aversive outcomes. In turn, these unrelenting negative reflections become habitual and can insidiously contribute to depressive symptoms. 

## 5. Recognizing Perfectionists

As the above discussion illustrated, distinguishing high achieving individuals from perfectionists who are vulnerable to depressive symptoms is not straightforward. For therapists, three key psychological instruments are available to measure perfectionism: the Frost Multidimensional Perfectionism Scale (FMPS), developed by Randy Frost and colleagues; the Hewitt Flett Multidimensional Perfectionism Scale (HFMPS), developed by Paul Hewitt and Gordon Flett; and the Almost Perfect Scale-Revised (APS-R), developed by Robert Slaney. 

Frost et al.'s [1] Multidimensional Perfectionism Scale (FMPS) addresses setting high standards and critical self-evaluation. The scale contains 35 items yielding six subscales. These subscales measure the six factors considered to be characteristic of perfectionists: Concern over Mistakes (9 items reflecting negative reactions to errors), Personal Standards (7 items indicating setting high standards for evaluation), Organization (6 items reflecting the importance placed on orderliness), Parental Expectations (5 items indicating beliefs that parents set very high standards), Parental Criticism (4 items reflecting belief that parents were overly critical), Doubt about Actions (4 items indicating tendencies to doubt ability). Cronbach's alpha for all of the FMPS subscales ranged from  .77 to  .93 indicating acceptable levels of internal consistency in a sample of female college students [1]. The FMPS is available from Dr. Randy Frost, Smith College Mass, USA.

Hewitt and Flett's [11] Multidimensional Perfectionism Scale (HFMPS) measures different aspects or factors of perfectionism. The scale contains 45 items measuring the intrapersonal and interpersonal dimensions of perfectionism: Self-Oriented Perfectionism (unrealistic standards and perfectionistic motivation for the self), Other-Oriented Perfectionism (unrealistic standards and motivations for others), and Socially Prescribed Perfectionism (believing others expect one to be perfect). The factor structure was congruent across clinical and subclinical populations. Alpha coefficients were  .88 (self-oriented perfectionists), 74 (other-oriented perfectionists), and  .81 (socially prescribed perfectionists) in a sample of psychiatric patients. Used in a later study with a sample of unipolar depressed patients, the HFMPS revealed that depressed patients were distinguished by a higher level of self-oriented perfectionism and that the depressed groups had higher levels of socially prescribed perfectionism than control groups [6]. The HFMPS must be purchased from Dr.'s Paul Hewitt and Gordon Flett through Multi-Health Systems, NY, USA.

While the FMPS and the HFMPS focus on maladaptive dimensions of perfectionism, Slaney et al.'s [17] 23 item Almost Perfect Scale-Revised (APS-R) was developed to measure adaptive as well as maladaptive elements of perfectionism. The APS-R measures: High Standards (how important doing one's best is), Order (how important being organized and neat is), and Discrepancy (the extent to which one feels that one's performance is meeting one's expectations) [17]. The Discrepancy subscale also measures maladaptive perfectionism by assessing the degree of chronic separation between high standards and eventual outcomes. For example, “I am seldom able to meet my own high standards for performance” [17]. Thus, the APS-R also measures critical self-evaluation and emphasizes the personal aspects of negative perfectionism in order to provide a stronger basis for assessment and clinical intervention. Alpha coefficients were  .85 (high standards),  .68 (order), and .92 (discrepancy) in a sample of college students. The APS-R is available from Dr. Slaney, Penn State University, PA, USA.

For nontherapists and the lay public, Gordon Flett offered a set of questions to shed light on behaviours that begin to signal maladaptive perfectionism. Unlike the FMPS, HFMPS, and APS-R, which were designed for therapists, Flett's set of questions are readily available to the public and require no specialized training. The questions are as follows.

*Top Ten Signs Your a Perfectionist

1. You can not stop thinking about a mistake you made.
2. You are intensely competitive and can't stand doing worse than others.
3. You either want to do something “just right” or not at all.
4. You demand perfection from other people.
5. You will not ask for help if asking can be perceived as a flaw or weakness.
6. You will persist at a task long after other people have quit.
7. You are a fault-finder who must correct other people when they are wrong.
8. You are highly aware of other people's demands and expectations.
9. You are very self-conscious about making mistakes in front of other people.
*You noticed the error in the title of this list [45].

These important questions, framed from a light-hearted perspective, provide nurses with a useful starting point for understanding how accepting only perfection in self and others is likely to lead to emotional distress. Nurses, like many professionals who work in exacting environments where mistakes can be deadly, are not immune to maladaptive perfectionism themselves. Given that as many as two thirds of some university student samples were categorized as perfectionistic [25], that self-critical perfectionism was positively correlated with depression in a sample of both premedical and non-premedical students [46] and that strong associations between perfectionism and psychological distress were found in a sample of medical, dental, nursing, and pharmacy students [47], perfectionist personality styles can be expected among nurses.

### 5.1. Recognizing Perfectionism on a Personal Level

Recognizing perfectionism on a personal level is important for nurses' own health. Perfectionist tendencies such as overassuming responsibility to ensure tasks are completed flawlessly [13] leave nurses exhausted and unfulfilled. Tendencies to make excuses about why actions will not be perfect rather than to risk acting [13] leave nurses powerless and unable to learn new tasks. Tendencies to shun situations where imperfections might be displayed [45] leave nurses isolated and unsupported. Perhaps most disturbing of all however, is that tendencies to keep problems to oneself and not admit failures to others [45] leave nurses ill prepared to disclose mistakes they do make. Rather than receiving recognition for their strivings or help when they need it, maladaptive nurse perfectionists can find themselves struggling within their relationships and becoming increasingly vulnerable to symptoms of depression.

Relationships with family, friends, and colleagues suffer when nurses either demand perfection from significant others or believe that those significant others will accept only perfection. Maladaptive perfectionists who are especially depression prone have often experienced perfectionist parenting themselves [4]. When parents communicate that their affection and approval is conditional on good performance, children can develop perfectionist personality styles that persist throughout adulthood [48]. Overly demanding and critical parents' model obsessive concern with mistakes [48]. In adulthood, nurses from perfectionist primary families may expect the same critical judgement from authority figures and from those who are important to them. They may also perpetuate the cycle of perfectionism with their own children. Identifying that one may have come from a family where self-worth is contingent only on consistent achievement is an important first step in recognizing perfectionism on a personal level. 

 Further, recognizing perfectionism-related depression in the workplace is another important consideration for nurses' own health. Socially prescribed perfectionism, or believing that important others will evaluate one harshly, is a vulnerability factor in the experience of burnout, job dissatisfaction, and depression [49]. In one study simulating performance situations where participants received failure feedback, perfectionism was a vulnerability factor in elevated dysphoric affect and negative cognition [50]. Perfectionist individuals are particularly sensitive to feedback indicating that their performance is not perfect, and when they believe others view their work as substandard, they experience intense feelings of decreased self-worth. Perfectionist leaders can contribute to feelings of burnout and negativity among members of their team, particularly with those who have a perfectionist personality style themselves. 

### 5.2. Recognizing Perfectionism on a Professional Level

Clients who are vulnerable to perfectionist-related depression will present in all areas of nursing practice. Coping with developmental milestones, acute or chronic health issues, and caring for others can be expected to exacerbate strivings to cope perfectly. Ineffective coping is prevalent among maladaptive perfectionists [51]. When faced with disability or chronic illness, scores from one sample group of maladaptive perfectionists were significantly high for negative adjustment and above the clinical cutoff for depression [52]. As nurses engage and motivate clients to strive for excellence in managing health, recognizing their perfectionist personality styles and the depressive symptoms that are likely to accompany them will strengthen care. 

When clients' ways of coping fall short of health care providers' expectations, perfectionists are likely to experience heightened feelings of being evaluated harshly. Subsequently, these demoralizing feelings could result in avoiding health care providers, not admitting to health issues, and declining much needed help. On initial assessment, a connection between clients' avoidance behaviors and seemingly limited motivation to change may not immediately signal potentially maladaptive perfectionism. And yet, recognizing when clients' strivings for excellence in managing their health care are actually manifesting as clinically significant perfectionism is critical. Follow-up assessments for symptoms of depression are indicated. Psychiatric or psychological referrals may also be indicated. As our understanding of the association between perfectionism and depression deepens, problems with the notion of accepting only perfection in self, colleagues, and clients become apparent.

## 6. Balancing Strategies

Shifting habitual patterns of thinking away from focusing on past or future mistakes and towards new ways of thinking will take time. Cognitive behavioral therapy with a psychiatric or psychological health professional could take months or even years. When comorbid conditions such as major depression or suicidal ideation are also present, therapeutic approaches become more complex. It is beyond the scope of this article to elaborate on comprehensive treatment of depression. However, the process of creating balancing strategies to differentiate striving for excellence from maladaptive striving for perfectionism begins with recognizing perfectionist personality styles in self, family, colleagues, and clients. Once individuals are willing to acknowledge that potential problems with perfectionist tendencies exist, they can begin to reimagine new and more balanced ways of looking at their previous accomplishments and future goals. Strategies for decreasing maladaptive perfectionist thinking will help lessen the feelings of decreased self-worth associated with depression. 

For self-oriented perfectionists, or those who require perfection in themselves, self-help books offer important direction. For example, in *Feeling Good: The New Mood Therapy* Burns' [53] chapter titled: *Dare to Be Average! Ways to Overcome Perfectionism* offers useful self-assessment tools and action-oriented suggestions for identifying cognitive distortions, and then replacing them with more rational ways of thinking. In *It's Your Little Red Wagon: 6 Six Core Strengths for Navigating Your Path to the Good Life*, Esonis [54] encourages readers to accept that they can be excellent, but not perfect, at some chosen goals, and mediocre at others. She invites readers to make decisions about selecting endeavours that merit their finest efforts and to plan to celebrate accomplishments—even those that were not achieved fully. In* Be Happy Without Being Perfect: How to Worry Less and Enjoy Life More*, Domar and Kelly [55] provide suggestions, especially for women, for focusing less on perceived failures or mistakes and more on successes and meaningful relationships. 

For other-oriented perfectionists, or those who require perfection in others, Marano's [48] advice, although geared primarily to parents, guides individuals to acknowledge efforts others put forward more than results they obtain. For example, rewarding family, employees', or clients' own process of dealing with an issue rather than the product or outcome they achieve projects genuine encouragement. Rather than offering external material awards, Marano [48] suggests that parents ask their children about why things went well and what they attribute their success to. Rather than expressing disappointment in completion of a task, Marano [48] encourages parents to ask children how they feel about their performance and what they might do differently next time. The same process of inquiry can be transferred to communication with adults. Parents, employers, and health professionals may not realize their demands for perfection are actually undermining performance and generating feelings of failure in those they are seeking to help. Once again, recognizing that perfectionist tendencies exist and they impact others is a key balancing strategy.

For socially-prescribed perfectionists, or those who believe important others in their life will accept only perfection, realistic assessment is essential. In instances where parents, employers, or health professionals are in fact other-oriented perfectionists, a belief that others are demanding perfection is likely credible. Here, strategies for dealing with people who are in positions of “power-over” are useful. Identifying clear specific areas where improvement is required and creating a step-by-step plan for changing behaviour is in order. Including designated times for reporting progress and discussing any further action required by the person in authority is warranted. 

However, when careful assessment reveals that others are not actually demanding perfection, strategies for minimizing self-handicapping, or spending more time finding excuses for poor performance than preparing for a good performance [13], are called for. Fearing harsh evaluation and making mistakes of any sort, socially-prescribed perfectionists avoid opportunities for the very evaluation from others that could improve their performance. Here, strategies for seeking safe appraisals from those who are perceived to be personable and supportive as well as credible offer important balance. For example, teachers and coaches as well as parents can contribute to children's feelings of success. So, ensuring that children have a variety of opportunities to learn new skills and a range of adults who offer them feedback is valuable. Likewise, self, peer, and client evaluations can supplement employees' performance appraisals. In the workplace, seeking opportunities for evaluative input that extends beyond immediate supervisors is useful. In sport, learning and practice environments, seeking extra drill, lab or simulation time can help reduce performance anxiety. In environments where clients believe health professionals demand perfection, attending support groups where others share what has worked for them and what has not is beneficial. 

Coping with life's inevitable failures is not easy for those whose self-worth is contingent on success. Perfectionists' deep-rooted habits of avoiding any situation where they might fail and ruminating over even inconsequential mistakes may have been present since childhood. At the heart of establishing balance and decreasing perfectionists' vulnerability to depression is learning to handle failure. As Marano [48] states: “Success hinges less on getting everything right than on how you handle getting things wrong” (page 86). 

One strategy for changing an ingrained habit is to create an interruption. For example, watching an engaging movie or play can interrupt ruminative thinking. Even when the interruption is short-lived, a fresh perspective becomes possible. Following an interruption, perfectionist thinking can shift away from mistakes and towards concrete plans for change. Listening mindfully to music, playing games, and visiting friends can create interruptions and divert thinking. Days off and vacations can also interrupt ruminative thinking, and perfectionists may need to put specific plans in place to ensure that they can let go of professional responsibilities during these times. In the same way, attending to nutritious eating, appropriate exercise, and pleasant time with loved ones are other healthy lifestyle strategies that perfectionists can implement to help create balance. 

Finally, intentionally replacing negative patterns of thinking with pleasurable experiences can be as simple as making time to focus on natural wonders. Moving out of a familiar room and into another space to look at a sunset or listen to a rainfall can feel invigorating. Spending time outdoors and finding ways to connect with plants and animals can provide brief respite from the relentless burdens of perfectionism. 

## 7. Conclusion

Individuals with perfectionist personality styles are vulnerable to symptoms of depression, especially when their maladaptive perfectionism becomes clinically significant. Ingrained habits of accepting nothing less than perfection in self and others can leave people of all ages feeling worthless and even suicidal. While healthy perfectionist tendencies foster success, the patterns of concern over mistakes, self-doubt, and expectation of criticism inherent in unhealthy perfectionist tendencies cause distorted thinking and social isolation. 

Explorations of perfectionism as a vulnerability factor in depression have been underway for several decades now and sufficient evidence exists to warrant nurses' attention and concern. Recognizing when strivings for excellence have shifted into strivings for absolute perfection is critical. Depression-prone perfectionism may be reflected in behaviour such as consistently setting unrealistic goals, ruminating, never feeling satisfied with one's own or others' performance, avoiding evaluation by important others, and declining needed help. Nurses themselves, their families, their workplace colleagues, and their clients are all susceptible to maladaptive perfectionism. 

Strategies for achieving balance include recognizing perfectionist personality styles in self and others, attending psychological or psychiatric therapy if necessary, acting on suggestions from self-help books, attending support groups, seeking evaluative feedback and help from a variety of different sources, learning to deal with failure, interrupting ruminative thought patterns, and looking for ways to intentionally replace negative thoughts with positive ideas. As nurses continue to learn about the links between perfectionism and symptoms of depression, more opportunities for nurturing excellence without exacerbating perfectionist tendencies will emerge. 
